# Factors Related to Participation in Health Examinations for Japanese National Health Insurance: NIPPON DATA2010

**DOI:** 10.2188/jea.JE20170251

**Published:** 2018-03-05

**Authors:** Haruhiko Imamura, Mana Kogure, Yoshikuni Kita, Hideaki Nakagawa, Atsushi Hozawa, Tomonori Okamura, Yoshitaka Murakami, Nobuo Nishi, Nagako Okuda, Aya Kadota, Takayoshi Ohkubo, Hirotsugu Ueshima, Akira Okayama, Katsuyuki Miura

**Affiliations:** 1 Department of Environmental and Occupational Health, School of Medicine, Toho University, Tokyo, Japan; 2 Department of Preventive Medicine and Epidemiology, Tohoku Medical Megabank Organization, Tohoku University, Miyagi, Japan; 3 Faculty of Nursing Science, Tsuruga Nursing University, Fukui, Japan; 4 Medical Research Institute, Kanazawa Medical University, Ishikawa, Japan; 5 Department of Preventive Medicine and Public Health, Keio University School of Medicine, Tokyo, Japan; 6 Department of Medical Statistics, School of Medicine, Toho University, Tokyo, Japan; 7 International Center for Nutrition and Information, National Institute of Health and Nutrition, National Institutes of Biomedical Innovation, Health and Nutrition, Tokyo, Japan; 8 Department of Health and Nutrition, University of Human Arts and Sciences, Saitama, Japan; 9 Center for Epidemiologic Research in Asia, Shiga University of Medical Science, Shiga, Japan; 10 Department of Public Health, Shiga University of Medical Science, Shiga, Japan; 11 Department of Hygiene and Public Health, Teikyo University School of Medicine, Tokyo, Japan; 12 Research Institute of Strategy for Prevention, Tokyo, Japan

**Keywords:** health examination, Japanese National Health Insurance, NIPPON DATA2010

## Abstract

**Background:**

This study investigated relationships among socioeconomic factors and participation in health examinations for Japanese National Health Insurance (NHI) using a representative Japanese population.

**Methods:**

We used the linkage database of NIPPON DATA2010 and Comprehensive Survey of Living Conditions 2010. Participants with NHI aged 40–74 years were included in the analysis. Prevalence ratios (PRs) for participation in health examinations in the past year were set as an outcome. Participant characteristics, including sex, age, socioeconomic factors (educational attainment, employment, equivalent household expenditure [EHE], house ownership, and marital status), laboratory measures, and lifestyle were included in an age-stratified modified Poisson regression analysis to examine relationships.

**Results:**

The number of study participants was 812, and 564 (69.5%) participated in health examinations in the past year. Among those aged 40–64 years, there was no significant PR for socioeconomic factors. Among those aged 65–74 years, high (≥13 years) educational attainment (adjusted PR, 1.22; 95% confidence interval [CI], 1.05–1.41) and house ownership (PR 1.40; 95% CI, 1.11–1.77) were positively associated with participation, while high (4th quartile) EHE (PR 0.84; 95% CI, 0.73–0.97) was negatively associated.

**Conclusion:**

These results suggest that high educational attainment, house ownership, and low EHE were positive factors for participation in health examinations among those aged 65–74 years.

## INTRODUCTION

Japan initiated the universal health care insurance system in 1961.^1^ Although there are approximately 3,500 insurers, they may be roughly divided into two groups: community-based and employee-based.^1^^–^^3^ A representative of the former is municipal National Health Insurance (NHI), which is managed by local municipalities and has the most insured persons (approximately 36 million insured persons in 2010^2^), consisting mainly of self-employed workers, farmers, part-timers, and retired persons aged 74 years or younger. The latter includes Association-Managed Health Insurance (for employees in large companies) and Society-Managed Health Insurance (for employees in small-to-medium companies). A feature of the system is the equality of the co-payment rate for medical care, except for the elderly and children.^1^ Furthermore, there is an Advanced Elderly Medical Service System, which covers all people aged 75 years and older.

Previous studies suggested that participation in health examinations was associated with lower mortality.^4^^–^^7^ Therefore, health care insurers conduct health examinations for insured persons. For example, from April 2008, all insurers were obliged to conduct specific health check-ups (examination), which are part of the national health service system targeting all insured persons aged 40–74 years for the purpose of the prevention and early detection of metabolic syndrome.^8^ However, participation rates in health examinations differed among health care insurers. In municipal NHI, the participation rate in specific health check-ups was the lowest among all health insurers (32.0% in the municipal NHI vs 43.2% among all insurers in 2010).^9^ Therefore, improvements in the participation rate are an important issue from a public health perspective, particularly for the NHI.

Previous studies reported factors for increasing the participation rate in health examinations. Regarding socioeconomic factors and social factors, social participation,^10^^,^^11^ social support or contact,^10^^–^^14^ and marital status (had a spouse)^15^ were positively associated with participation in health examinations directly or indirectly. Furthermore, health consciousness,^12^ a good subjective health condition,^15^ self-efficacy,^14^ and health knowledge^14^ were positive factors for participation. In contrast, medical facility visits^12^ and low instrumental activities of daily living (IADL)^12^ were negatively associated with participation. To the best of our knowledge, nationwide data has not yet been used to investigate relationships among socioeconomic factors and participation in health examinations in consideration of objective health indicators, such as laboratory measurements and health behaviors.

The purpose of the present study is to investigate relationships among socioeconomic factors and participation in health examinations among NHI-insured persons using nationwide cross-sectional data with comprehensive items, including laboratory measurements and health behaviors.

## METHODS

### Study population

We used data from the National Integrated Project for Prospective Observation of Non-communicable Disease and its Trends in the Aged 2010 (NIPPON DATA2010) and Comprehensive Survey of Living Conditions 2010 (CSLC2010).^16^ NIPPON DATA2010 was conducted as the National Health and Nutrition Survey of Japan 2010 (NHNS2010), which surveyed residents stratified and randomly selected from CSLC2010 participants and consisted of a dietary intake survey, lifestyle survey, and physical examination, including a blood test. NIPPON DATA2010 and NHNS2010 were conducted in November 2010 and CSLC2010 was conducted in June 2010. NHNS2010 and CSLC2010 were implemented by the Ministry of Health, Labour and Welfare of Japan. In CSLC2010, 289,363 households from 5,510 stratified and randomly selected census enumeration areas throughout Japan were surveyed and 229,785 households (79.4%) responded.^17^ These 5,510 census enumeration areas were then divided into unit blocks, which each consisted of 20–30 households. In NHNS2010, 300 unit blocks (5,357 households) were randomly selected and 3,684 households (68.8% of 5,357), which consisted of 8,815 participants, responded.^17^ Among participants who were 20 years or older, 3,873 completed blood tests^17^ and 2,891 subsequently responded to the survey for NIPPON DATA2010, which included additional blood tests and a questionnaire related to cardiovascular diseases, with informed consent.

**Figure 1.**
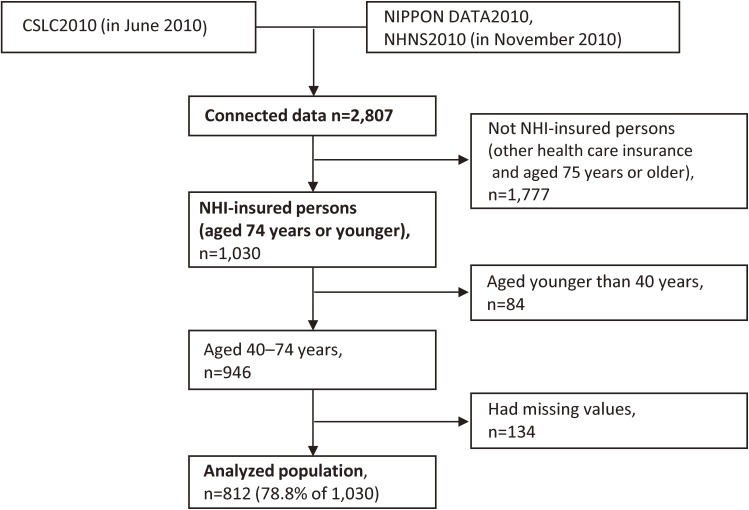
Study population: NIPPON DATA2010. CLCS2010, Comprehensive Survey of Living Conditions 2010; NHI, National Health Insurance; NHNS2010, National Health and Nutrition Survey of Japan 2010; NIPPON DATA2010, National Integrated Project for Prospective Observation of Non-communicable Disease and its Trends in the Aged 2010.

We successfully connected 2,807 participants’ data (97.1% of 2,891) with the data of CSLC2010. Of those, 1,030 participants were NHI-insured persons who were 74 years or younger (1,777 were insured by other health care insurance; 751 were insured under employee-based insurance such as Association-Managed Health Insurance and Society-Managed Health Insurance, 468 were dependent on employee-based insurance, 455 were insured under Advanced Elderly Medical Service System, and 103 were insured by other insurance or unknown). Among these eligible study participants, we excluded those who were younger than 40 years (*n* = 84) and those who had missing values (*n* = 134). Therefore, 812 participants (78.8% of 1,030) aged 40–74 years were analyzed in this study (Figure 1). This study was approved by the Institutional Review Board of Shiga University of Medical Science (No. 22-29, 2010).

### Outcome measurement

The outcome measurement was “participation in the past year in health examinations” assessed by the following question in CSLC2010: “Have you participated in a health examination (such as a specific health check-up, health examination conducted by municipalities, and comprehensive medical examination) in the past year?” Response alternatives were “Yes” or “No”. There was a supplementary explanation stating that “the health examination in this question does not include screening for cancer only, a maternal examination, dental health examination, or medical examination for a diagnosis at a hospital or clinic”. The answer of “Yes” was treated as participation.

### Exploring factors

In addition to age and sex, educational attainment, employment, equivalent household expenditure (EHE; calculated as household monthly expenditure, which did not include health care insurance premiums or housing loans, divided by the square root of the number of members per household), house ownership, and marital status were selected as socioeconomic factors; body mass index (BMI; calculated as weight divided by the square of height), systolic blood pressure (average of 2 measurements), HbA1c (National Glycohemoglobin Standardization Program [NGSP]), and total cholesterol as blood test results related to health conditions; outpatient care (whether under treatment as an outpatient or not) and instrumental activities of daily living (IADL) as health conditions; and smoking habits, alcohol habits, and exercise habits as health behaviors. Sex, age, educational attainment, BMI, systolic blood pressure, HbA1c, total cholesterol, IADL, smoking habits, alcohol habits, and exercise habits were obtained from NIPPON DATA2010 and NHNS2010, while other variables were obtained from CSLC2010.

Systolic blood pressure, HbA1c, and total cholesterol were divided into three categories in combination with the assessment of medication (antihypertensive drugs, insulin or hypoglycemic drugs, and cholesterol-lowering drugs, respectively) as follows: “normal value without medication”, “high value without medication”, and “medication”. The definitions of “high value” were as follows: systolic blood pressure was 140 mm Hg or higher, HbA1c was 6.5% or higher, and total cholesterol was 220 mg/dL or higher.

Other variables, except for age, were divided into two to four categories: sex (“men” and “women”); educational attainment (“<10 years”, “10–12 years”, and “≥13 years”); employment (“full time” and “other [part time job, housekeeping, and student]”); EHE (four quartiles of all connected data); house ownership (“no house ownership” and “house owner”); marital status (“married” and “widowed, divorced, or single”); BMI (“<18.5 kg/m^2^”, “18.5–21.9 kg/m^2^”, “22–24.9 kg/m^2^” and “≥25 kg/m^2^”); outpatient care (“no/unknown” and “yes”); IADL (“≥11 points” and “<11 points” out of 13 measured by the Tokyo Metropolitan Institute of Gerontology Index of Competence, which measures higher-level competence, such as instrumental self-maintenance, intellectual activity, and social roles, with 13 items^18^); exercise habits (“none for health reasons”, “none for other reasons”, and “exercise”); smoking habits (“never”, “ex-smoker”, and “current smoker”); and alcohol habits (“never”, “ex-drinker”, and “current drinker”). The answer of “unknown” in the “outpatient visit” was included in “not visit” category because only 11 participants answered “unknown”. The cut-off value for IADL was set based on the average value of Japanese people older than 65 years.^19^

### Statistical analysis

The prevalence ratio (PR) for participation in health examinations was calculated using a multivariable-adjusted modified Poisson regression analysis^20^ stratified by age: 40–64 years and 65–74 years. This stratification accounts for the constituents of NHI changing at an age of approximately 65 years, which is the retirement age. Each socioeconomic factor was included in the model adjusting for age and sex (model 1). All sixteen factors were then included in the model (model 2). The significance level was set at *P* < 0.05. All analyses were performed with STATA version 14.0 (STATA Corporation, College Station, TX, USA).

## RESULTS

The mean age of the study population (*n* = 812) was 64.5 (standard deviation [SD], 7.8) years. Of those, 46.2% were men and 564 (69.5%) participated in health examinations in the past year. Table 1 shows the characteristics of the study population. The mean age of the 40–64 years group (*n* = 323) was 56.8 (SD, 6.7), and 44.6% were men. The mean age of the 65–74 years group (*n* = 489) was 69.5 (SD, 2.8), and 47.2% were men.

**Table 1.** Characteristics of the study population: NIPPON DATA2010

	40–64 years (*n* = 323)	65–74 years (*n* = 489)
**Sex**		
men	144 (44.6%)^a^	231 (47.2%)
women	179 (55.4%)	258 (52.8%)
**Age**		
mean (SD) years	56.8 (6.7)	69.5 (2.8)
**Educational attainment**		
<10 years	63 (19.5%)	170 (34.8%)
10–12 years	175 (54.2%)	231 (47.2%)
≥13 years	85 (26.3%)	88 (18.0%)
**Employment**		
full time	138 (42.7%)	92 (18.8%)
other	185 (57.3%)	397 (81.2%)
**Equivalent household expenditure**		
1st quartile	104 (32.2%)	114 (23.3%)
2nd quartile	84 (26.0%)	123 (25.2%)
3rd quartile	68 (21.1%)	114 (23.3%)
4th quartile	67 (20.7%)	138 (28.2%)
**House ownership**		
no house ownership	48 (14.9%)	56 (11.5%)
house owner	275 (85.1%)	433 (88.5%)
**Marital status**		
married	254 (78.6%)	407 (83.2%)
widowed, divorced, or single	69 (21.4%)	82 (16.8%)
**BMI**		
<18.5 kg/m^2^	16 (5.0%)	20 (4.1%)
18.5–21.9 kg/m^2^	106 (32.8%)	137 (28.0%)
22–24.9 kg/m^2^	96 (29.7%)	195 (39.9%)
≥25 kg/m^2^	105 (32.5%)	137 (28.0%)
**Systolic blood pressure**		
normal value without medication (<140 mm Hg)	159 (49.2%)	175 (35.8%)
high value without medication (≥140 mm Hg)	92 (28.5%)	115 (23.5%)
medication	72 (22.3%)	199 (40.7%)
**HbA1c (NGSP)**		
normal value without medication (<6.5%)	279 (86.4%)	409 (83.6%)
high value without medication (≥6.5%)	28 (8.7%)	25 (5.1%)
medication	16 (5.0%)	55 (11.2%)
**Total cholesterol**		
normal value without medication (<220 mg/dL)	154 (47.7%)	234 (47.9%)
high value without medication (≥220 mg/dL)	131 (40.6%)	140 (28.6%)
medication	38 (11.8%)	115 (23.5%)
**Outpatient care**		
no/unknown	185 (57.3%)	159 (32.5%)
yes	138 (42.7%)	330 (67.5%)
**IADL**		
≥11 points	306 (94.7%)	454 (92.8%)
<11 points	17 (5.3%)	35 (7.2%)
**Exercise habits**		
none for health reasons	10 (3.1%)	31 (6.3%)
none for other reasons	206 (63.8%)	209 (42.7%)
exercise	107 (33.1%)	249 (50.9%)
**Smoking habits**		
never	193 (59.8%)	323 (66.1%)
ex-smoker	58 (18.0%)	120 (24.5%)
current smoker	72 (22.3%)	46 (9.4%)
**Alcohol habits**		
never	131 (40.6%)	231 (47.2%)
ex-drinker	5 (1.5%)	10 (2.0%)
current drinker	187 (57.9%)	248 (50.7%)

BMI, body mass index; IADL, instrumental activities of daily living; NGSP, national glycohemoglobin standardization program; SD, standard deviation.

^a^Proportion of *n* for each age category.

Table 2 shows relationships among participation in health examinations and socioeconomic factors in the 40–64 years group. In this group, 198 (61.3%) participated in health examinations in the past year. Regarding each socioeconomic factor, the highest and lowest prevalence of participation in health examinations were as follows; “women” (67.0%) and “men” (54.2%) in sex, “10–12 years” (64.6%) and “≥13 years” (56.5%) in educational attainment, “other” (64.9%) and “full time” (56.5%) in employment, “1st quartile” (63.5%) and “3rd quartile” (57.4%) in EHE, “house owner” (63.3%) and “no house ownership” (50.0%) in house ownership, “married” (64.6%) and “widowed, divorced, or single” (49.3%) in marital status. In the multivariable-adjusted modified Poisson regression analysis (model 2), there was no significance in socioeconomic factors. There were also no significant differences in results depending on models.

**Table 2.** Relationships among participation in health examinations and related factors in the 40–64 years group: NIPPON DATA2010

(*n* = 323)						

	Outcome/Study Population (%)		Model 1^a^		Model 2^b^	
	
			PR (95% CI)^c^	*P*-Value	PR (95% CI)	*P*-Value

**Sex**						
men	78/144	(54.2%)	1		1	
women	120/179	(67.0%)	1.23 (1.03–1.47)	0.03	1.23 (0.96–1.59)	0.10
**Age**						
(continuous)	—	—	1.02 (1.00–1.03)	0.03	1.01 (0.99–1.02)	0.35
**Educational attainment**						
<10 years	37/63	(58.7%)	1		1	
10–12 years	113/175	(64.6%)	1.13 (0.90–1.43)	0.29	1.17 (0.96–1.43)	0.13
≥13 years	48/85	(56.5%)	1.05 (0.79–1.39)	0.74	1.06 (0.82–1.35)	0.67
**Employment**						
full time	78/138	(56.5%)	1		1	
other	120/185	(64.9%)	1.00 (0.82–1.21)	0.99	1.02 (0.84–1.23)	0.88
**Equivalent household expenditure**						
1st quartile	66/104	(63.5%)	1		1	
2nd quartile	53/84	(63.1%)	0.97 (0.78–1.20)	0.76	0.91 (0.75–1.10)	0.33
3rd quartile	39/68	(57.4%)	0.87 (0.68–1.10)	0.24	0.80 (0.63–1.01)	0.06
4th quartile	40/67	(59.7%)	0.93 (0.73–1.19)	0.56	0.90 (0.69–1.17)	0.44
**House ownership**						
no house ownership	24/48	(50.0%)	1		1	
house owner	174/275	(63.3%)	1.20 (0.89–1.62)	0.23	1.09 (0.83–1.43)	0.55
**Marital status**						
married	164/254	(64.6%)	1		1	
widowed, divorced, or single	34/69	(49.3%)	0.79 (0.62–1.01)	0.07	0.86 (0.68–1.10)	0.23


BMI, body mass index; CI, confidence interval; IADL, instrumental activities of daily living; PR, prevalence ratio.

^a^Model 1. Adjusted for age (continuous) and sex for each factor.

^b^Model 2. Adjusted for age (continuous), sex, and all other related factors (educational attainment, employment, equivalent household expenditure, house ownership, marital status, BMI, systolic blood pressure, HbA1c, total cholesterol, outpatient care, IADL, exercise habits, smoking habits, and alcohol habits).

^c^Adjusted PR and 95% CI were calculated by a modified Poisson regression analysis.

Table 3 shows the relationships among participation in health examinations and socioeconomic factors in the 65–74 years group. In this group, 366 (74.8%) participated in health examinations in the past year. Regarding each socioeconomic factor, the highest and lowest prevalence of participation in health examinations were as follows: “women” (77.1%) and “men” (72.3%) in sex, “≥13 years” (81.8%) and “<10 years” (69.4%) in educational attainment, “other” (75.3%) and “full time” (72.8%) in employment, “1st quartile” (80.7%) and “4th quartile” (70.3%) in EHE, “house owner” (77.4%) and “no house ownership” (55.4%) in house ownership, “married” (75.7%) and “widowed, divorced, or single” (70.7%) in marital status. In the multivariable-adjusted modified Poisson regression analysis (model 2), PRs for “≥13 years of educational attainment” (adjusted PR, 1.22, 95% CI, 1.05–1.41 in model 2) and house ownership (adjusted PR, 1.40; 95% CI, 1.11–1.77 in model 2) were significantly high, whereas “4th quartile of EHE” (adjusted PR 0.84; 95% CI, 0.73–0.97) was low. There were no significant differences in results depending on models.

**Table 3.** Relationships among participation in health examinations and related factors in the 65–74 years group: NIPPON DATA2010

(*n* = 489)						

	Outcome/Study Population (%)		Model 1^a^		Model 2^b^	
	
			PR (95% CI)^c^	*P*-Value	PR (95% CI)	*P*-Value

**Sex**						
men	167/231	(72.3%)	1		1	
women	199/258	(77.1%)	1.07 (0.96–1.18)	0.22	1.11 (0.96–1.28)	0.15
**Age**						
(continuous)	—	—	1.00 (0.98–1.02)	0.78	1.00 (0.98–1.02)	0.82
**Educational attainment**						
<10 years	118/170	(69.4%)	1		1	
10–12 years	176/231	(76.2%)	1.10 (0.97–1.24)	0.15	1.12 (0.99–1.27)	0.07
≥13 years	72/88	(81.8%)	1.19 (1.03–1.37)	0.02	1.22 (1.05–1.41)	0.01
**Employment**						
full time	67/92	(72.8%)	1		1	
other	299/397	(75.3%)	1.02 (0.88–1.17)	0.83	0.98 (0.85–1.14)	0.82
**Equivalent household expenditure**						
1st quartile	92/114	(80.7%)	1		1	
2nd quartile	96/123	(78.0%)	0.98 (0.86–1.11)	0.71	0.99 (0.87–1.12)	0.82
3rd quartile	81/114	(71.1%)	0.89 (0.77–1.03)	0.11	0.89 (0.77–1.03)	0.13
4th quartile	97/138	(70.3%)	0.88 (0.76–1.01)	0.07	0.84 (0.73–0.97)	0.02
**House ownership**						
no house ownership	31/56	(55.4%)	1		1	
house owner	335/433	(77.4%)	1.40 (1.10–1.78)	0.01	1.40 (1.11–1.77)	0.01
**Marital status**						
married	308/407	(75.7%)	1		1	
widowed, divorced, or single	58/82	(70.7%)	0.92 (0.79–1.07)	0.26	0.96 (0.83–1.12)	0.63


BMI, body mass index; CI, confidence interval; IADL, instrumental activities of daily living; PR, prevalence ratio.

^a^Model 1. Adjusted for age (continuous) and sex for each factor.

^b^Model 2. Adjusted for age (continuous), sex, and all other related factors (educational attainment, employment, equivalent household expenditure, house ownership, marital status, BMI, systolic blood pressure, HbA1c, total cholesterol, outpatient care, IADL, exercise habits, smoking habits, and alcohol habits).

^c^Adjusted PR and 95% CI were calculated by a modified Poisson regression analysis.

There were no significant interactions between age group (40–64 and 65–74 years) and each socioeconomic factor (in the crude model in which other factors were not included).

## DISCUSSION

Our results showed that educational attainment, EHE, and house ownership were related factors for participation in health examinations in the 65–74 years group, while these relationships were not found in the 40–64 years group. In the 65–74 years group, PR for “≥13 years of educational attainment” and house ownership were high, while “4th quartile of EHE” was low.

Previous studies reported that high educational attainment was positively associated with health and health behaviors.^21^^,^^22^ Furthermore, house renters were less healthy than house owners because house ownership reflected the accumulation of income and wealth during the life course.^21^ The results of this study on educational attainment and house ownership were consistent with previous findings for participation in health examinations.

In addition, EHE, which may be an alternative of equivalent income, was also regarded as a potential related factor. Previous studies showed that a high income was positively associated with health and health behaviors.^21^^–^^23^ However, PR was low in the “4th quartile (highest EHE)” group in this study. A high income has been negatively associated with health behaviors in Asian regions.^15^^,^^24^ Since the fee for a specific health check-up (examination)^8^ is free for persons from low-income households in Japan, they may be more likely to participate in health examinations. Further studies are needed to confirm this.

There were no significant differences by sex, employment, or marital status in the 40–64 years and 65–74 years groups. Regarding sex, our results were consistent with previous findings.^11^^,^^15^ As for marital status, which may represent an aspect of social support, a previous study showed that having a spouse was positively associated with participation.^15^ In this study, the prevalence of participation was slightly higher in “married” subjects than in those who were “widowed, divorced, or single”.

Although there was no significant interaction, relationships were observed among socioeconomic factors and participation in health examinations in the 65–74 years group, but not in the 40–64 years group. This result may be attributed to the weaker influence of socioeconomic factors than other factors, such as health conditions and health behaviors, in the 40–64 years group. Since studies on socioeconomic health differences among the elderly are relatively new,^21^ the results of this study suggest the effects of socioeconomic factors on health behaviors in the elderly.

Our study has two major strengths. We used a nationwide linkage database. Furthermore, many factors for participation in health examinations were comprehensively investigated. In particular, laboratory blood tests were used in this study. To the best of our knowledge, this is the first study to investigate objective health indicators as adjustment factors for participation.

Although we used a nationwide database in the present study, there were concerns that our study participants did not represent all NHI-insured persons. The prevalence of participation in health examinations among study participants was 69.5%, whereas it was 32.0% among NHI participants in 2010. This suggests that study participants were very health conscious, so these results need to be interpreted carefully. Our study results indicate that the prevalence of participation varied depending on socioeconomic factors after adjusting for laboratory measures and lifestyles, particularly in the 65–74 years group. These results also suggest the necessity of socioeconomic measures for those at risk of deteriorating health.

In conclusion, high educational attainment, low EHE, and house ownership were related factors for participation in health examinations in the 65–74 years group.
